# The Treatment of Intestinal Obstruction

**Published:** 1897-07-03

**Authors:** 


### THE TREATMENT OF INTESTINAL OBSTRUCTION.

The treatment of intestinal obstruction in its varying phases will always possess a paramount interest for the practitioner, for among the many serious and urgent conditions that he is called upon to treat this occurs with considerable frequency, and often with but little warning. The advances in abdominal surgery, and our rapidly enlarging acquaintance with the pathological conditions concerned in abdominal diseases, have caused treatment by drugs to fall almost into complete desuetude, and operative interference to assume a prominence which 15 or 20 years ago would have been opposed to the opinions of large numbers in the profession, and which even now elicits at rare intervals an occasional protest from some, whose experience and reputation invest it with peculiar weight.

In these anxious cases the object of the surgeon's endeavours is to obtain a cure of the disease where that is possible, and to relieve or to palliate when, from the conditions known to exist, cure is out of the question. In malignant disease palliative operations for long held the field, but the proportion of cases in which cure is aimed at by the removal of the growth is rapidly increasing, and the point to which this latest development can be pushed without turning to the disadvantage of the patient is one upon which much divergence of opinion is likely to arise.

But in the non-malignant cases, especially those in which the symptoms are of a more or less acute character, it is by no means certain that the best lines of treatment are clearly established. In such cases exploratory abdominal section is practically the rule, and probably most men would say that the proper course is to find the cause of the obstruction and remove it. And in due time this will be so.

But the surgeon's most important duty is so to pilot his patient in his course that in avoiding the Charybdis of obstruction he does not wreck him upon the Scylla of operation shock. In no class of case is the judgment and wisdom of the operator so put to the test. To search for and find the seat and cause of the obstruction in an abdomen stretched to its utmost by distended intestine, and in a patient whose powers of resistance are reduced to the lowest point as a result of several days' pain, vomiting, sleeplessness, and starvation, and whose very existence could not be much longer maintained, is an operation frequently prolonged, always most severe, and sometimes impossible.

The operation is in a large number of instances the last straw—and what wonder? The wonder is that such cases sometimes recover.

But this severe handling of the patient is only absolutely necessary in a proportion of the cases. If, before operating, the diagnosis can be made between obstruction and strangulation—and in most cases it can, if sufficient pains be taken—the case in which everything must be risked to set free the strangled bowel can be separated from that in which the relief of obstruction only is required to save life. The decision of the surgeon on this one point may prove to be of enormous importance to the patient. If he believes that he has to deal with a strangulation, then he can only hope to save his patient by its immediate release; but if the condition is one of obstruction not complicated with strangulation, then he has two courses to choose between. Either he may do a complete operation—*i.e.*, find and remove the cause—or he may content himself with opening a distended coil and relieving the obstruction, leaving the cause of the mischief to be dealt with at a later date, when the patient's condition and powers of resistance have largely recovered, and when the intra-abdominal conditions have assumed a form more suitable for exploratory manipulations.

There can be no question as to which is the least serious measure to adopt. Equally certain is it that the high mortality following complete operations is largely dependent upon the severity of the procedure practised on a patient whose vitality is at a low ebb.

The feeling is gradually growing that by dividing the complete operation into two stages, as already indicated, many cases may be saved who would infallibly be sacrificed if submitted to it in one. But the double operation has its drawbacks. It entails upon the patient a period of unpleasantness inseparable from an artificial anus and a raw abdominal surface, and upon the surgeon some disquietude at the thought that he will have to explore the abdomen through a surface very difficult to render aseptic. But the surgical difficulties are by no means insuperable, and the subsequent procedures, namely, finding and removing the obstruction, and closing the artificial anus may be undertaken with reasonable prospects of success.

Of course, there are many cases of obstruction in which the cause is quickly revealed upon exploration, and readily dealt with. It is not to these, however, to which we refer, but to that even larger group in which it is evident that, however skilful the manipulations, the effect upon the patient is sure to be such as to make his recovery a matter of extreme doubt, if they require more than a very short period for their performance.

The post-operative results of intestinal obstruction leave much to be desired, but the improvement which may be hopefully looked for in the future will be due as much to an improving judgment on the part of the surgeon as to increasing operative dexterity.

